# Accounting for relatedness in family-based association studies: application to Genetic Analysis Workshop 18 data

**DOI:** 10.1186/1753-6561-8-S1-S79

**Published:** 2014-06-17

**Authors:** Jakris Eu-ahsunthornwattana, Richard AJ Howey, Heather J Cordell

**Affiliations:** 1 Institute of Genetic Medicine, Newcastle University, Central Parkway, Newcastle upon Tyne, NE1 3BZ, UK; 2 Division of Medical Genetics, Department of Internal Medicine, Faculty of Medicine Ramathibodi Hospital, Mahidol University, Rama VI Rd, Ratchathevi, Bangkok 10400, Thailand

## Abstract

In the last few years, a bewildering variety of methods/software packages that use linear mixed models to account for sample relatedness on the basis of genome-wide genomic information have been proposed. We compared these approaches as implemented in the programs EMMAX, FaST-LMM, Gemma, and GenABEL (FASTA/GRAMMAR-Gamma) on the Genetic Analysis Workshop 18 data. All methods performed quite similarly and were successful in reducing the genomic control inflation factor to reasonable levels, particularly when the mean values of the observations were used, although more variation was observed when data from each time point were used individually. From a practical point of view, we conclude that it makes little difference to the results which method/software package is used, and the user can make the choice of package on the basis of personal taste or computational speed/convenience.

## Background

A number of different methods/software packages have been proposed in the last few years that implement linear mixed-model approaches to account for population structure and relatedness among samples in genome-wide association studies (GWAS), but no detailed comparisons among them have been made before our effort. Indeed, when a new method/package is developed, it is often quite unclear whether or how it differs substantially from those already available. To address this question, we explored the performance of various implementations of such methods in the longitudinal Genetic Analysis Workshop 18 (GAW18) data set.

## Methods

We analyzed the GAW18 GWAS data [1] using the real phenotypes and the first set of simulated phenotypes. This analysis was performed without knowledge of the underlying simulating model. The genotype data were cleaned using standard procedures [2]. This resulted in 4 individuals being excluded because of their total lack of genotype data, and another individual being excluded because of outlying ethnicity (Chinese [CHB] or Japanese [JPT]), leaving 954 individuals whose genotype data were used. We removed 43,987 monomorphic or low-frequency (minor allele frequency [MAF] <1%) single-nucleotide polymorphisms (SNPs), 109 SNPs with missing rate above 10% (this criterion took into account the apparently high missing rate in some SNPs likely to be caused by the differences in genotyping technology used in the samples), and 1 SNP that failed Hardy-Weinberg equilibrium testing in the control founder population. A total of 427,952 SNPs were retained for analysis.

We conducted linear regression of the real and simulated systolic blood pressure and simulated diastolic blood pressure at each time point regressed on age, medication, and smoking status. For the real diastolic blood pressure--which, as could be physiologically expected, seemed to have a nonlinear relationship with age--we used a quadratic regression, including age and age squared as predictors. The phenotype data from all individuals were used for these regressions. Residuals from these regressions in subjects who also had genotype data were then used for the genome-wide analyses.

Genome-wide association analyses, adjusting for familial relatedness using genomic data, were performed using a variety of linear mixed model approaches. All approaches attempt to fit the model ***Y***=*β*+*Q*+***ε***, where ***Y***=(*y*_1_, ..., *y_n_*)*^T ^*is a vector of responses on *n *subjects; *X*= (*x_ik_*) is the *n * × *K *matrix of predictor values for variables to be modeled as fixed effects (including covariates and genotypes at any SNPs currently under test); β=(β_1_, ... β_K_)*^T ^*are regression coefficients (to be estimated) representing the linear effects of the predictors on the response; *Q *are random effects, *Q*~N(0,2σ_g_^2^Φ), and ε are random errors, ε~N(0,σ_e_^2^*I*), where σ_g_^2 ^and σ_e_^2 ^are parameters (to be estimated) representing the genetic and environmental components of variance respectively; Φ is the *n * × *n *matrix of pairwise kinship coefficients; and *I *is the *n * × *n *identity matrix. The approaches vary with respect to precise details of the calculation of kinship or "relatedness" and with respect to whether an exact method or a fast approximation is used (for more details, see descriptions in references [3-9]). In each case we used a subset of 21,153 SNPs to perform the relatedness calculations, namely SNPs with MAF >0.4, <5% missing data, and "pruned" to be in approximate linkage equilibrium via the PLINK command "-indep 50 5 2". In analyses of other data sets we have found little difference between results when using such a pruned set of SNPs for calculating relatedness and when using the full set of SNPs (data not shown).

The methods considered were: (a) EMMAX [3], which implements 2 methods for relatedness calculations: one based on identity-by-state (IBS) sharing and one based on the Balding-Nichols method [4]; (b) FaST-LMM [5], which also implements 2 methods to adjust for relatedness: one using a standard covariance matrix and one using the realized relationship matrix; (c) the polygenic/mmscore functions in GenABEL [6], which implement the FASTA method [7]; (d) the polygenic/grammar functions in GenABEL, which implement the GRAMMAR-Gamma approximation [8]; and (e) Gemma [9], which uses an efficient exact method. Simple linear regression without any relatedness adjustment was also performed in FaST-LMM. All analyses were performed using both the residual from each individual observation (modeled without regard to its true longitudinal nature, or *longitudinal*) and the mean of the residuals for each subject, or *mean*. Genomic inflation factors (λ) were calculated as proposed by Devlin and Roeder [10]. We also assessed the genomic inflation factors for unadjusted χ^2 ^and Cochran-Armitage trend tests of hypertension status at each time point as calculated using PLINK [11].

## Results and discussion

**Figure 1:**
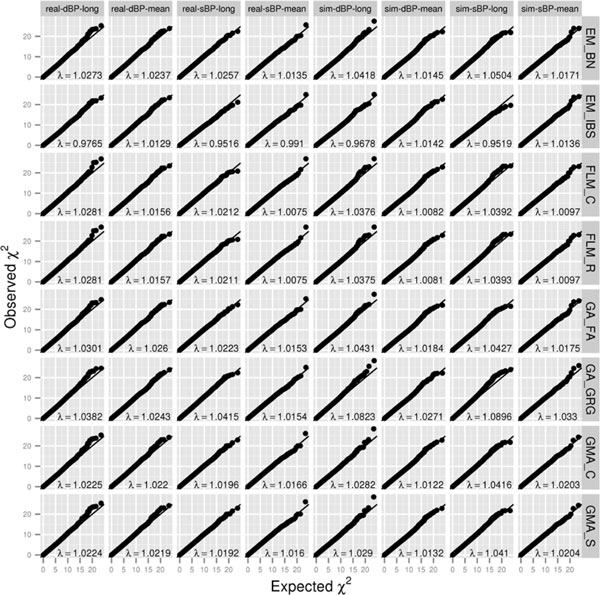
**Q-Q plots and genomic inflation factors for different methods**. These were calculated for each phenotype (real diastolic blood pressure [DBP], real systolic blood pressure [SBP], simulated DBP, and simulated SBP), using either longitudinal ("long") or average ("mean") residuals. EM_BN, EMMAX using Balding-Nichols matrix; EM_IBS, EMMAX using IBS matrix; FLM_C, FaST-LMM using standard covariance matrix; FLM_R, FaST-LMM using realized relationship matrix; GA_FA, GenABEL/FASTA; GA_GRG, GenABEL/GRAMMAR-Gamma; GMA_C, Gemma using centralized covariance matrix; GMA_S, Gemma using standardized covariance matrix. The diagonal line represents the identity line in each panel.

Figure 1 shows the Q-Q plots and genomic inflation factors for different methods. It is well known that population substructure and relatedness will cause an inflated distribution of genome-wide association test statistics (λ >1.00) if not appropriately modeled. All methods performed reasonably well for the mean residuals, controlling the λ to 0.99 to 1.03. For longitudinal data, most methods also performed well, with λ in the range of 0.95 to 1.05, except perhaps for GRAMMAR-Gamma, which achieved λs of approximately 1.08 to 1.09 for the simulated phenotypes. However, even these values were much less inflated compared to the λ values of 1.22 to 1.68 (mean) and 2.04 to 3.41 (longitudinal) seen in the unadjusted analyses. The higher inflation in longitudinal analyses (even when adjusting for relatedness) could be expected from the fact that additional (nongenetic) within-subject correlation was not allowed for in these analyses; indeed, one could argue that this behavior is statistically the "correct" behavior, with GRAMMAR-Gamma (which gave the highest inflation) showing the "most correct" behavior. Interestingly, EMMAX using the IBS matrix seemed to have the opposite behavior, for reasons we are currently unable to determine.

For the analyses using hypertension status, the unadjusted genomic inflations were between 1.21 and 1.55 for the Cochran-Armitage trend test and between 1.01 and 1.27 for the χ^2 ^test.

**Figure 2:**
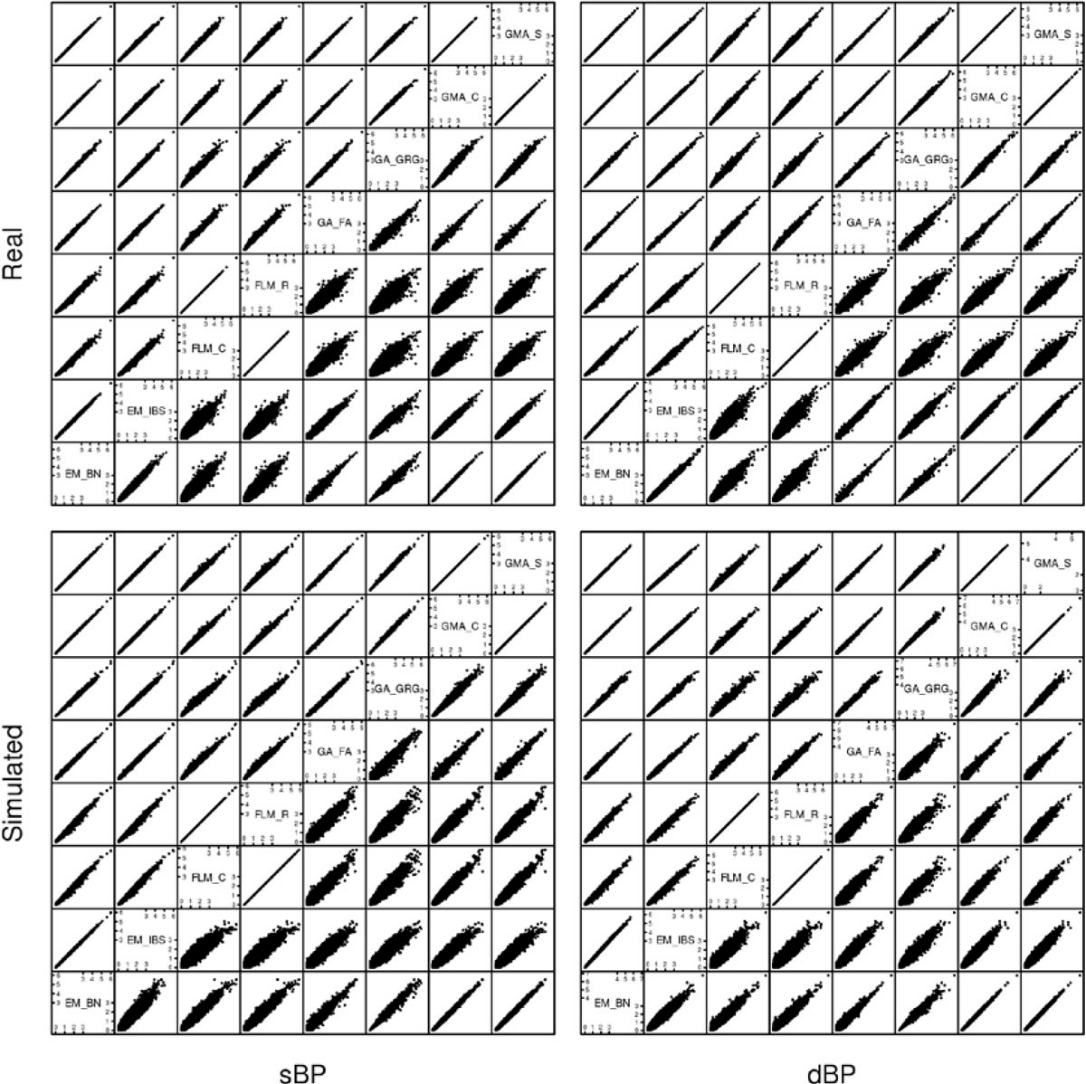
**Comparison of −log_10 _*p *values at each SNP calculated using different methods**. The upper triangles show the values based on mean residuals, while the lower triangles show the values calculated using longitudinal data. DBP, diastolic blood pressure; EM_BN, EMMAX using Balding-Nichols matrix; EM_IBS, EMMAX using IBS matrix; FLM_C, FaST-LMM using standard covariance matrix; FLM_R, FaST-LMM using realized relationship matrix; GA_FA, GenABEL/FASTA; GA_GRG, GenABEL/GRAMMAR-Gamma; GMA_C, Gemma using centralized covariance matrix; GMA_S, Gemma using standardized covariance matrix; SBP, systolic blood pressure.

Figure 2 compares the individual −log_10 _*p *values from different methods. Most methods gave highly concordant results, particularly EMMAX (BN) and Gemma, whereas the 2 GenABEL methods were similar but less concordant. This is analogous to findings on single-observation data by Zhou and Stephens [9]. FaST-LMM tended to perform slightly differently from the other methods at SNPs with lower significance, although the results overall were still quite similar.

**Figure 3:**
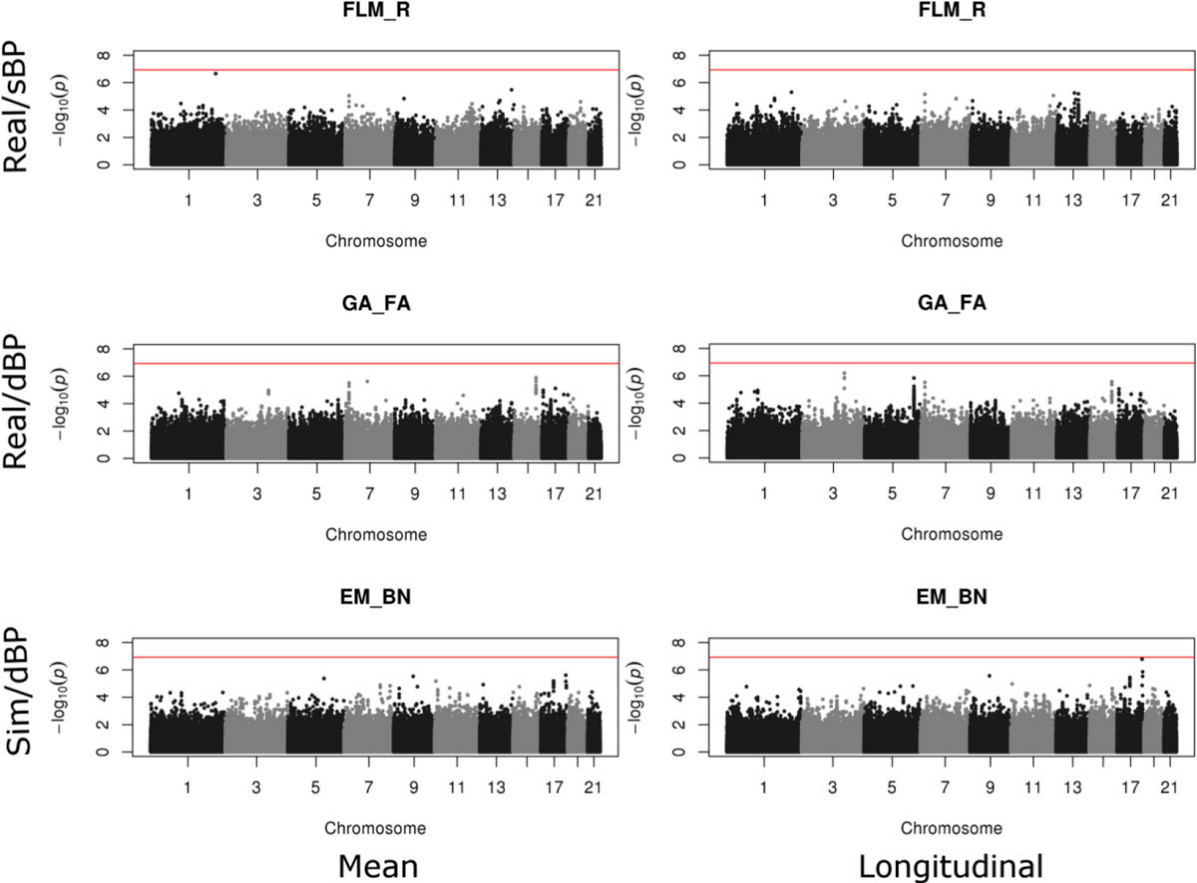
**A selection of Manhattan plots showing *p *values calculated using various methods**. DBP, diastolic blood pressure; EM_BN, EMMAX using Balding-Nichols matrix; FLM_R, FaST-LMM using realized relationship matrix; GA_FA, GenABEL/FASTA; SBP, systolic blood pressure.

Figure 3 shows a selection of Manhattan plots. For each phenotype, the results from all methods were quite similar, although the longitudinal data tended to show stronger signals. No clearly significant SNP was found in any phenotype, which is not surprising given the relatively small size of the GAW18 data set, which is underpowered for detecting (at genome-wide levels of significance) anything other than strong genetic effects. The high concordance in significance levels (at any given SNP) achieved by the different software packages (see Figure 2) indicates that no package is substantially more powerful than another, as expected from the fact that all packages implement slightly different versions of essentially the same statistical model.

Although the results from all packages considered here were similar, the implementations did vary in speed. All packages performed the analysis in reasonable time (less than 1 day) on our system. Precise timings will depend on the computer resources and architecture available, but as a rule of thumb we found FaST-LMM and GRAMMAR-Gamma to be the fastest (taking just a few hours), followed by EMMAX and Gemma, which took 12 to 16 hours, and GenABEL/FASTA, which took 18 to 20 hours.

## Conclusions

All methods performed well and results were similar, particularly at the most significant SNPs. We conclude that (at least for nonlongitudinal traits) it makes little difference to the results which method/software package is used, and the user can make the choice of package on the basis of personal taste, speed, or computational convenience. For longitudinal traits (modeled without regard to their longitudinal nature) the slight differences seen between the methods would be an interesting topic for further investigation, but it is beyond the scope of the current article.

## Competing interests

The authors declare that they have no competing interests.

## Authors' contributions

JE conducted the statistical analyses and drafted the manuscript. RAJH prepared the data and conducted statistical analyses. HJC conceived the overall study and critically revised the manuscript. All authors read and approved the final manuscript.
